# Mesohepatectomy with total caudate lobectomy of the liver for hepatocellular carcinoma

**DOI:** 10.1186/1477-7819-11-82

**Published:** 2013-04-04

**Authors:** Hiromichi Ishii, Shinpei Ogino, Koki Ikemoto, Atsushi Toma, Kenji Nakamura, Tsuyoshi Itoh, Toshiya Ochiai

**Affiliations:** 1 Division of Surgery, Kyoto Prefectural Yosanoumi Hospital, 481 Otokoyama, Yosano-cho, Yosa-gun, Kyoto, 629-2261, Japan

**Keywords:** Mesohepatectomy with total caudate lobectomy, Hepatocellular carcinoma, Hepatectomy

## Abstract

**Background:**

Mesohepatectomy with total resection of the caudate lobe and extrahepatic bile duct is sometimes performed for hilar cholangiocarcinoma or gallbladder carcinoma; however, only a few reports on mesohepatectomy with total caudate lobectomy of the liver for hepatocellular carcinoma are available.

**Methods:**

A 71-year-old woman was preoperatively diagnosed with hepatocellular carcinoma in the central bisections (Couinaud’s segments 4, 5, and 8) and the paracaval portion of the caudate lobe. Mesohepatectomy with total caudate lobectomy of the liver permitted the removal of tumors to provide a cancer-free raw surface of the liver. Mobilization of the caudate lobe is an important procedure in this surgery. Before the liver parenchyma was dissected, all short hepatic veins were ligated and divided from the left to the right side as the left lateral section was retracted to the right, and the caudate lobe branches of the portal vein and hepatic artery were ligated and divided. After the liver parenchymal dissection, both between the left lateral and medial sections and between the right anterior and posterior sections, the Glissonean branches of the caudate lobe were ligated and divided as the central bisections were anteriorly retracted. Finally, liver parenchymal dissection was performed between the caudate lobe and the right posterior section, which was along the right side of the inferior vena cava.

**Results:**

The surgery time was 538 minutes and blood loss was 1,207 mL. No blood transfusions were required during or after surgery. The postoperative course was uncomplicated. The patient is still alive 25 months after hepatectomy.

**Conclusion:**

Although mesohepatectomy with total caudate lobectomy of the liver is technically more difficult than mesohepatectomy of the liver because the caudate lobe must be completely detached from the inferior vena cava and the hilar plate, it is a safe and effective treatment method in selected patients with hepatocellular carcinoma located at both the central bisections and the paracaval portion of the caudate lobe.

## Background

The caudate lobe of the liver, which is located behind both major lobes and is surrounded by the inferior vena cava (IVC), three main hepatic veins, and hepatic hilum, is divided into Spiegel’s lobe, the paracaval portion, and the caudate process. The left lateral extremity of the paracaval portion is on the Arantius canal, the right lateral extremity is on the left of the right posterior portal vein, the cranial extremity crosses over the middle hepatic vein (MHV) and the right hepatic vein (RHV), and the caudal extremity is on the right portal vein. The portion to the left of Arantius canal is defined as Spiegel’s lobe, and the portion on the caudal side of the right portal vein is defined as the caudate process [1-3].

The indications for mesohepatectomy of the liver, which involves removal of Couinaud’s segments 4, 5, and 8 [4], include tumors occupying the left medial and right anterior sections [5]. If the tumor is located at the paracaval portion of the caudate lobe, total caudate lobectomy with removal of adjacent portions of the liver can be performed for patients with good liver function, and isolated total caudate lobectomy is recommended for patients with poor liver function [6]. Therefore, for patients with hepatocellular carcinoma (HCC), which is located mainly at the central bisections (Couinaud’s segments 4, 5, and 8) and extends to the paracaval portion of the caudate lobe, and with good liver function, mesohepatectomy with total caudate lobectomy of the liver is recommended.

Left or right hemihepatectomy with total caudate lobectomy is sometimes performed for patients with HCC originating from or invading the caudate lobe [7]; however, to the best of our knowledge, only a few reports on mesohepatectomy with total caudate lobectomy of the liver for HCC are available, and there is a lack of technical description of this surgical procedure in the literature. In this study, we describe the surgical technique, mesohepatectomy with total caudate lobectomy of the liver, for HCC.

## Methods

### Patient

**Figure 1:**
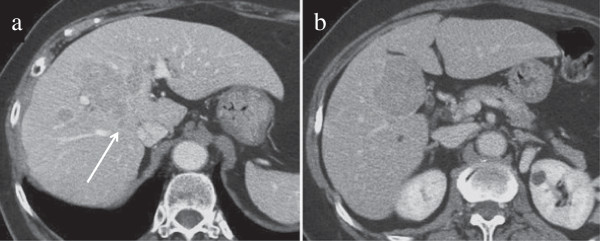
Computed tomography revealed a tumor located mainly at Couinaud’s segments 4, 5, and 8, and this tumor extended to the paracaval portion of the caudate lobe in the liver (arrow).

A 71-year-old woman was admitted to our hospital for treatment of liver tumors. Abdominal dynamic computed tomography (CT) revealed a lesion with high- and low-density areas located mainly at Couinaud’s segments 4, 5, and 8 in the arterial and venous phases, respectively, and this tumor extended to the paracaval portion of the caudate lobe in the liver (Figure 1a and 1b). The tumor was 5 cm in diameter and located near the root of the right anterior Glissonean pedicle. CT also revealed two daughter lesions in segments 4 and 8. The Child–Pugh classification status was class A (5 points). In addition, on the scoring system designed by the Liver Cancer Study Group of Japan [8] the degree of liver damage was given as class A. Both classifications were made on the basis of laboratory data obtained at the time of hospitalization (serum total bilirubin level, 0.5 mg/dL; serum albumin concentration, 4.6 g/dL; prothrombin activity, 93.9%; serum aspartate aminotransferase level, 40 IU/L; serum alanine aminotransferase level, 31 IU/L; and indocyanine green retention value at 15 minutes after intravenous injection (ICG-R15), 9%]. We preoperatively diagnosed the patient with stage II and III HCC according to the classifications of the International Union against Cancer [9] and the Liver Cancer Study Group of Japan [8], respectively, and decided to perform mesohepatectomy with total caudate lobectomy of the liver.

### Surgical technique

A right intercostal incision along the ninth intercostal space with an upper-midline extension was used to perform laparotomy and thoracotomy, and the round ligament was ligated and divided. We routinely performed intraoperative ultrasonography to define the tumor location and vessels to be manipulated for resection during hepatectomy. The left hemiliver was completely mobilized by dissecting the falciform, left coronary, and left triangular ligaments. To prevent the remaining right posterior section from being twisted after extraction of the liver, the right posterior section was not mobilized, that is, the bare area and right adrenal gland were not dissected, even though the right coronary and right triangular ligaments were dissected. The ventral surfaces of the RHV and MHV roots were exposed.

**Figure 2:**
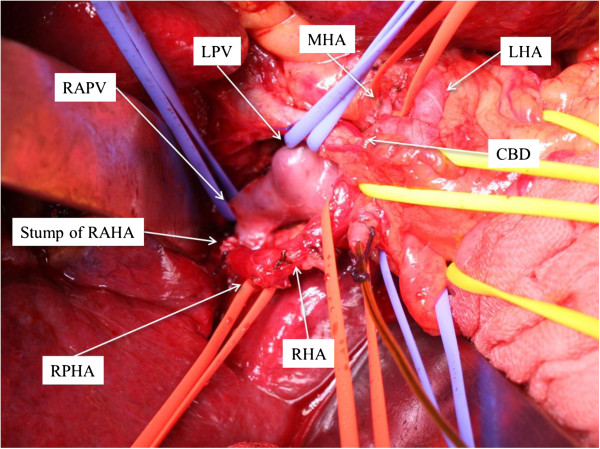
**The RHA, RAHA, RPHA, MHA, LHA, LPV, RPV, and RAPV were encircled and taped at the hepatic hilum before hepatic parenchymal transection.** CBD, common bile duct; LHA, left hepatic artery; LPV, left portal vein; MHA, middle hepatic artery; RAHA, right anterior hepatic artery; RAPV, right anterior portal vein; RHA, right hepatic artery; RPHA, right posterior hepatic artery; RPV, right portal vein.

A cholecystectomy was performed, and a 4-Fr biliary tube was inserted through the cystic duct to test bile leakage. The right, right anterior, right posterior, middle, and left hepatic arteries were encircled and taped at the hepatic hilum in sequence. Further, the roots of the right anterior and middle hepatic arteries and the caudate lobe branches of the hepatic artery were ligated and divided. Following exposure of the portal bifurcation, the caudate lobe branches of the portal vein, which joined the right and left portal veins were ligated and divided. The left, right, and right anterior portal veins were encircled and taped in sequence (Figure 2). The root of the right anterior portal vein was ligated without division because sufficient root length of the right anterior portal vein could not be exposed for division. Discoloration of the right anterior section was then confirmed. The demarcation line between the right anterior and posterior sections was marked by electric cautery.

The infrahepatic IVC at the cranial side of the left renal vein was encircled and taped so that it could be clamped during hepatic resection if the need arose.

**Figure 3:**
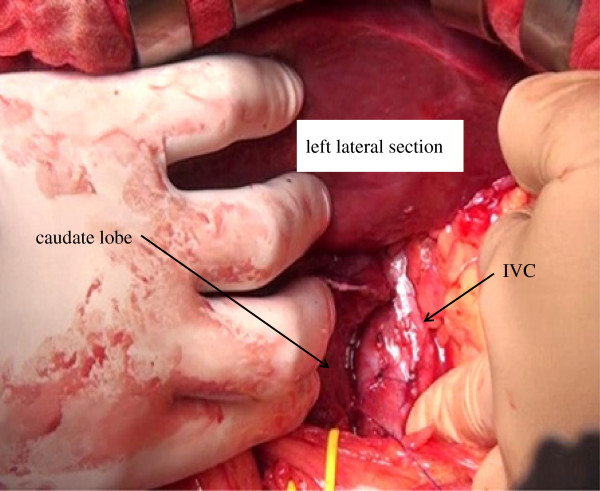
**Mobilization of the caudate lobe from the IVC was performed only from the left side.** IVC, inferior vena cava.

By retracting the left lateral section to the right, the lesser omentum was incised, and the Arantius canal was ligated and divided on the side of the left hepatic vein. Mobilization of the caudate lobe from the IVC was performed only from the left side, that is, all of the short hepatic veins were ligated and divided from the left toward the right and from the caudal toward the cranial directions following division of the left IVC ligament (Figure 3). The inferior right hepatic vein was preserved.

Transection of the hepatic parenchyma between the left lateral section and the left medial section was performed along the right side of the falciform ligament using the ultrasonic surgical aspirator under left hemihepatic vascular occlusion [10], and the Glissonean branches of Couinaud’s segment 4 were intrahepatically ligated and divided. At the cranial end of the hepatic parenchymal dissection, the MHV root was identified, and the MHV was divided at its origin using a vascular stapler. The hepatic parenchyma was then divided toward the Arantius canal.

Transection of the hepatic parenchyma between the right anterior and posterior sections was performed along the demarcation line from the caudal toward the cranial direction under right hemihepatic vascular occlusion. In the dissection plane depth, the left wall of the RHV was identified, and the branches of the RHV draining from the right anterior section were ligated and divided. After the hepatic parenchymal transection was performed toward the hepatic hilum, the right anterior portal vein was adequately exposed and divided. Further, the right anterior sectional branch of the bile duct was distally ligated and divided to avoid biliary injury of the right posterior sectional branch.

**Figure 4:**
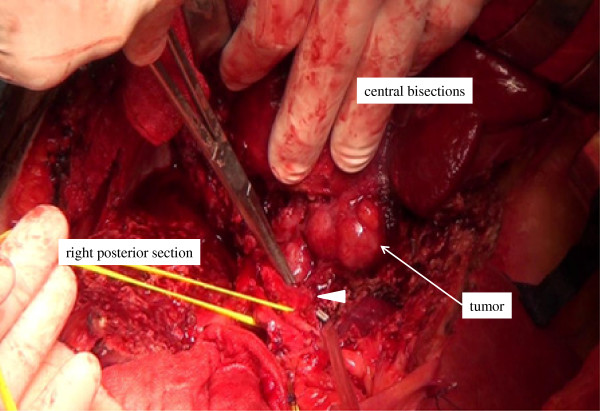
The Glissonean branch of the caudate lobe (arrow head) was ligated and divided under direct vision during the final hepatic parenchymal transection stage.

**Figure 5:**
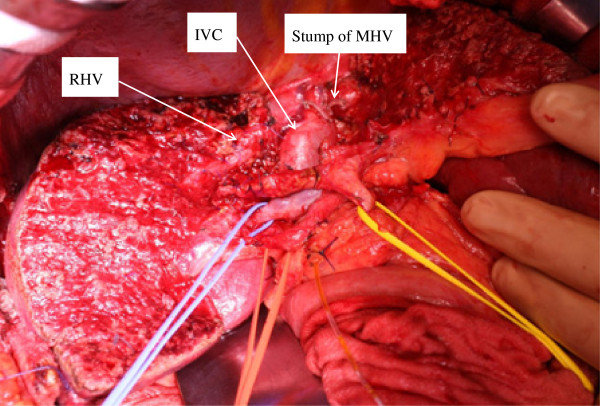
**After mesohepatectomy and total caudate lobe were removed, the RHV was exposed on the raw surface of the liver and the IVC was exposed.** IVC, inferior vena cava; RHV, right hepatic vein.

Detachment of the caudate lobe from the hilar plate was performed, and all Glissonean branches of the caudate lobe were ligated and divided by retracting the central bisection upward (Figure 4). Although the tumor was visible because the tumor was located near the surface of the liver, the tumor was not exposed on the raw surface of the liver (Figure 4). Following the complete exposure of the left wall of the RHV, the direction of hepatic parenchymal transection was changed toward the right edge of the IVC, which is the landmark between the caudate lobe and the right posterior section, under guidance of the surgeon’s left index finger that was inserted along the right edge of the IVC [11]. Mesohepatectomy with total caudate lobectomy was thereby complete (Figure 5).

The round ligament was fixed to the abdominal wall to prevent twisting of the remaining left lateral section.

The surgical duration was 538 minutes, and blood loss was 1,207 mL. No blood transfusions were required during or after surgery.

Microscopically, the tumor was a moderately differentiated HCC with tumor-negative surgical margins.

**Figure 6:**
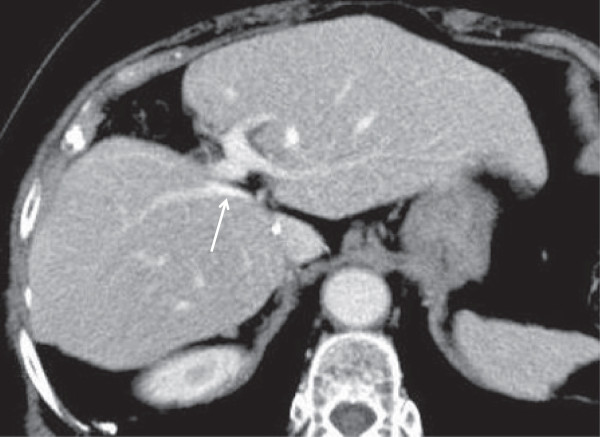
**Postoperative CT revealed that the central bisections and caudate lobe are removed, the RHV (arrow) is exposed on the raw surface of the liver, and the IVC is exposed.** IVC, inferior vena cava; RHV, right hepatic vein.

The postoperative course was uncomplicated. Postoperative peak levels of serum aspartate aminotransferase, alanine aminotransferase, and total bilirubin were 740 IU/L, 664 IU/L, and 1.0 mg/dL, respectively, and these levels returned to normal 11 days after surgery. The patient was discharged 13 days after the surgery. Postoperative CT revealed that the central bisections and caudate lobe are removed, the RHV is exposed on the raw surface of the liver, and the IVC is exposed (Figure 6). The patient is still alive 25 months after hepatectomy.

## Discussion

Mesohepatectomy [12-14] of the liver, which involves removal of Couinaud’s segments 4, 5, and 8, is not described in the Brisbane 2000 system of nomenclature of hepatic anatomy and resections [15]. The procedure is also referred to as central bisectionectomy [5,16], central hepatectomy [17], and central bisegmentectomy [18,19] in the literature.

Mesohepatectomy with total resection of the caudate lobe and extrahepatic bile duct is sometimes performed for hilar cholangiocarcinoma or gallbladder carcinoma [3,19-21]; however, to the best of our knowledge, there is only one report of mesohepatectomy with total caudate lobectomy of the liver without resection of the extrahepatic bile duct for HCC [22], and the surgical technique for this procedure has not been reported in detail.

Total caudate lobectomy is recommended for patients with HCC located at the paracaval portion of the caudate lobe to improve prognosis [6,22]. Therefore, mesohepatectomy with total caudate lobectomy may be reasonable for patients with HCC located at both the central bisections and the paracaval portion of the caudate lobe as in our case, or for patients in whom HCC that is located at the paracaval portion of the caudate lobe invades the MHV [22], if the patient’s liver function permits, that is, Child–Pugh classification status is A and ICG-R15 is <20% [13].

Sakoda *et al*. [22] reported that the remaining right posterior section gets twisted after mesohepatectomy with total caudate lobectomy blocking the RHV outflow because the right posterior section is completely mobilized. Therefore, we think that the right posterior section should not be mobilized to prevent twisting after liver extraction. A left-side approach should be used to mobilize the caudate lobe. Because the cranial side of the caudate lobe can be mobilized under good operative view by mobilization of the left lateral section, we mobilized the left lateral section in the present case. The round ligament was fixed to the abdominal wall to prevent twisting of the remaining left lateral section.

In mesohepatectomy, the extrahepatic or intrahepatic Glissonean pedicle transection method or dissection method of the hepatic artery, portal vein, and bile duct is usually performed [23,24], and the Glissonean pedicle transection method is preferable to avoid postoperative lymphatic leaks in patients with liver cirrhosis. In our case, the dissection method of the hepatic artery, portal vein, and bile duct was performed because the patient did not have liver cirrhosis, and we were more familiar with this method than with the Glissonean pedicle transection method. Although the caudate lobe branches of the portal vein and hepatic artery were ligated and divided before hepatic parenchymal transection, the Glissonean branches of the caudate lobe should be ligated and divided under direct vision during the final hepatic parenchymal transection stage. Mesohepatectomy or total caudate lobectomy is an independent risk factor for postoperative bile leakage because the cut liver surface exposes the Glisson’s sheath of the hepatic hilum [25,26]; therefore, meticulous procedures are necessary to avoid damage to the hepatic hilar bile duct.

It was reported that during hepatectomy, infrahepatic IVC occlusion was associated with significantly reduced intraoperative blood loss [27,28]. In our case, the infrahepatic IVC was encircled and taped; however, we did not clamp the infrahepatic IVC because it may have caused hemodynamic instability. Low central venous pressure achieved by anesthesiological interventions such as fluid restriction has been widely accepted to reduce intraoperative hemorrhage [29]. In our case, fluid infusion was restricted during parenchymal transection by the anesthesiologist; however, central venous pressure was not monitored because a central venous catheter was not used routinely.

With advances in surgical techniques and instruments, laparoscopic liver resection has been performed more frequently; however, only a few reports on laparoscopic mesohepatectomy [30,31] or caudate lobectomy [32] are available because they are technically demanding surgeries. Therefore, we think that laparoscopic mesohepatectomy with total caudate lobectomy should be performed by surgeons with expertise in both liver surgery and laparoscopic techniques.

## Conclusions

Although mesohepatectomy with total caudate lobectomy is a technically difficult surgery and requires special attention to prevent surgical complications, it is a safe and effective treatment method in selected patients with HCC located at both the central bisections and the paracaval portion of the caudate lobe.

## Consent

Written informed consent was obtained from the patient for publication of her clinical details and accompanying images. A copy of the written consent is available for review by the Editor of this journal.

## Abbreviations

CT: computed tomography; HCC: hepatocellular carcinoma; ICG-R15: indocyanine green retention value at 15 min after intravenous injection; IVC: inferior vena cava; MHV: middle hepatic vein; RHV: right hepatic vein.

## Competing interests

The authors declare that they have no competing interests.

## Authors’ contributions

HI wrote the first draft of this report. HI and TI performed the operation. HI, SO, KI, AT, KN, and TI performed the postoperative management. TO supervised the writing of the paper. HI is the guarantor of the paper. All authors read and approved the final manuscript.
